# Immunologic aspects of patients with disseminated bacille Calmette-Guerin disease in north-west of Iran

**DOI:** 10.1186/1824-7288-35-42

**Published:** 2009-12-23

**Authors:** Mahnaz Sadeghi-Shanbestari, Khalil Ansarin, Seyed Hudieh Maljaei, Mandana Rafeey, Zakaria Pezeshki, Ahmmad kousha, Reza Baradaran, Jean Laurent Casanova, Jacqueline Feinberg, Jean Pierre de Villartay

**Affiliations:** 1 TB and Lung Research Center of Tabriz (0098), Iran; 2 Pediatric Department, Division of immunology and Allergy, Children's Hospital, Tabriz University of Medical Sciences, Tabriz (0098), Iran; 3 Internal medicine Department, Emam Reza Hospital, Tabriz University of Medical Sciences, Tabriz (0098), Iran; 4 Faculty of Medicine, Tabriz University of Medical Sciences, Tabriz (0098), Iran; 5 Pediatric Department, Children's Hospital, Tabriz University of Medical Sciences, Tabriz (0098), Iran; 6 Sina Hospital, Tabriz University of Medical Sciences, Tabriz (0098), Iran; 7 The Laboratory of Human Genetics of Infectious Diseases, INSERM U.550, Necker Medical School, University Paris René Descartes, Paris, France, EU

## Abstract

**Background:**

Adverse reactions induced by BCG vaccination are rare, disseminated mycobacterial BCG infection in particular, which is often fatal and results from impaired immunity. The aim of this study is to determine the nature of the immunodeficiences in patients with disseminated BCG infection in northwest region of Iran.

**Materials and methods:**

Through 2 years all infants with BCG adenitis or other complications of this vaccine that had suspicious BCG infection were referred to children's hospital and health centers of Tabriz.

Evaluation of immune system and in some cases genetic survey was performed in infants with evidence of histopathologic demonstration of acid-fast bacilli. Then frequency of infants who had disseminated BCG infection with immunodeficiency was defined.

**Results:**

From 48 selected infants with complications of BCG vaccine in the range of 2 to 62 months, 28 infants (58.3%) were male and 20 infants (41.7%) were female. Disseminated BCG infection was diagnosed in 11 cases, almost all of whom had immunodeficiency as follows:

Seven cases had severe combined immunodeficiency and one cases had chronic granulomatous disease. MSMD in two cases and IL12 R deficiency in another one was diagnosed.

Overall, the mortality rate was 72.8% (8 cases) which 7 cases of them were SCID and another one CGD

Consanguineous was found in more than half (7 cases) of patients and family history of disseminated BCG infection or immunodeficiency was found in nearly one third (3 cases) of patients.

**Discussion:**

BCG vaccine is administered world wide to prevent tuberculosis and is considered to have excellent safety profile. However in some immunodeficient patients it can cause severe and fatal complications, like in our region, where all cases of disseminated BCG infection with severe immunodeficiency died.

**Conclusion:**

BCG vaccination is necessary in some countries such as Iran, so it seems that development of a more safer vaccine and change of vaccine program in the families with history of inherited immunodeficiency can be identifies such high risk infants and prophylaxis of severe complications or dead in such patients.

## Introduction

In Iran BCG sub strain Pasteur vaccine, is administered to all the newborns at birth to prevent tuberculosis. Adverse reactions induced by BCG vaccination are rare (ranging from zero to 23.8%) [1,2]. The most frequent complications are purulent regional lymphadenitis (0.9 per 1000 vaccinated children) [2]. Bone BCG infection is the second most frequent (0.39 to 46 per million vaccinated children) [3-5]. Disseminated infections are even rarer and their estimated incidence is 0.1 to 4.3 per one million vaccinated children but is lethal in 50 to 71% of the cases [6-8]. The death rate is especially higher in cases of immunodepression (83%) and it is important that a temporary or permanent immune deficiency observed in 86% of the cases [6-9]. Disseminated BCG infections have occurred in children with immunodeficiency disorders such as severe combined immunodeficiency (SCID), chronic granulomatous disease (CGD), complete Di George syndrome, AIDS and idiopathic immunodeficiency of genetic origin or mendelian susceptibility to mycobacterial disease(MSMD) with underlying genetic defects, but only rarely in apparently normal individuals[6,8-10].

The aim of this study was to determine the role of immunodeficiency disorders in exciting disseminated BCG infection.

## Materials and methods

This prospective study was performed through 24- month period by TB and lung disease research center of Tabriz because of in Iran all children at birth immunize with BCG vaccine. So, Children with BCG lymphadenitis and criteria as follows were referred to children's hospital from health centers by convenience sampling.

Inclusion criteria were: Lymphadenitis, Abscesses or fistula in the site of BCG vaccination or another site or BCG ulceration with 2 or more than of following such as:

1. Fever >38°c more than 2 weeks

2. Anemia (Hb<10)

3. Recurrent or persistent oral Candida

4. Organomegaly (Hepatosplenomegaly)

5. Bone diseases (Pain or arthritis)

6. Weight loss

7. Recurrent or persistent diarrhea

8. Related parents

9. Family history of immunodeficiency

Then physical examination and laboratory studies were performed based on:

1. A systemic syndrome compatible with mycobacterial disease. Typical manifestations include fever, weight loss, anemia and death.

2. Evidence of infection by positive acid-fast bacilli on smears and growth of M. bovis, BCG strain at two or more anatomic sites beyond the region of vaccination.

The competency of the immune system were evaluated by different tests including measurement of Igs, Isohemaglotination test, phagocyte activity by NBT (slide test), T and B cell counts by flowcytometry, delayed type hypersensitivity test (DHT). HIV performed by Elisa test. These tests were performed by standard procedures and by trained laboratory personnel in the specialized laboratories.

Clinical manifestations and hematological and immunologic changes were compared between two different groups of cases: Patients (who are children with disseminated BCG infection) and control group (who are children with complications of BCG vaccination but without disseminated BCG infection). Then frequency of patients with disseminated BCG infection who had immunodeficiency was reported.

The children and their parents were informed about the necessary clinical and laboratory examination procedures and their consent was taken before the start of the study.

### Static

Sings, symptoms and laboratory findings were compared between patients and control by Chi-square test and fisher exact test. Also, we used student t-test for comparation between T, B, NK cells and SPSS 14 for data analysis.

## Results

In this survey from 122093 BCG vaccinated children through 24 month period,48 infants with BCG lymphadenitis, within range of 2-62 months (mean ± SD: 9.9 ± 9.85) were selected. 28 infants were male (58.3%) and 20 infants were female (41.7%).

Onset age was between 1-21 months (mean ± SD: 6.36 ± 4.62). Twenty four cases had multiple lymphadenitis near to BCG incubation such as cervical, axiliary, and supraclaviculs, 11 cases had suppurative lymphadenitis with fistula and abscesses and 5 cases had ulcer of BCG incubation. Osteomyelitis due to BCG vaccination was detected in 2 cases and disseminated BCG infection in 11 cases (22.9%) with multi organ involvement and systemic symptoms was detected. Family history of BCG infection or immunodeficiency was positive in 22.9% of control group and the rate of consanguineous marriages in the parents of them was 37.5% (18 cases).

A chi-square test comparing the clinical manifestations in children with disseminated BCG infection with control group showed statistically significant differences for the two groups of children (Table 1).

**Table 1:** Comparison of clinical and laboratory findings in patients and control groups

Clinical and laboratory findings	Patient Number (%)	Control Number (%)	P. value
Lymphadenopathy	5(45)	10(27)	0.28
Organomegaly	5(45)	0	<0.001
Bone involvement	3(27.3)	1(2.7)	0.033
Lung involvement	9(81.8)	3(8.1)	<0.001
Thymus atrophy	9(81.8)	0	<0.001
Recurrent diarrhea	8(72.7)	9(24.3)	0.009
Recurrent infection	8(72.7)	3(8.1)	<0.001
Oral Candida	9(81.8)	5(13.5)	<0.001
Fever	11(100)	15(41.7)	0.005
Lymphopenia	8(72.7)	3(8.1)	<0.001
Anemia	11(100)	19(51.4)	0.003

Definitive immunodeficiency was detected in approximately all of children with disseminated BCG infection including: SCID in 7 cases with homozygous mutation and homozygous polymorphism in Rag2 in one patient with heterozygous for the Rag2 mutation and polymorphism in the both parents of him and exclusion of Rag1, Rag2 and Artemis defect in another patients.

Other cases were CGD, IL12RB1 deficiency and MSMD. In one cases of MSMD we could exclude an IL12RB1 deficiency. So he had a normal expression of IL12RB1on cell surface with two different antibodies. HIV was not identified in any of the cases (Table 2).

**Table 2:** summary of data on children with disseminated BCG infection

Number	Age/sex	Site(s) of dissemination	Immune defect	Outcome	Consanguinity	Family history immunodeficiency
1	9 mo/M	Chest, Spleen	MSMD	Survived	-	
2	3year/M	As cite, CNS Bone	CGD	died	-	
3	4 mo/M	DLN, Liver	SCID Rag_2 _deficiency T-B-NK+	died	+	+
4	4 mo/M	Bone, Pericardia Liver	Omen syn T+B-NK+	died	+	
5	6 mo/F	Gastric aspirate, DLN	T-B-NK+ SCID	died	-	
6	5 mo/F	DLN, Liver	T-B-NK+ SCID	died	+	+
7	4/5 mo/M	Eye, DLN, Lung (Miliary)	*T-B-NK+ SCID	died	+	
8	5 mo/F	As cite, Liver, DLN	SCID T-B+NK+	died	+	
9	3.5 years/M	Bone, Skin, DLN	MSMD	Survived	-	+
10	4 mo/F	DLN, Gastric aspiration	IL_12 _R deficiency	Survived	+	
11	4 mo/F	Urine, Liver	SCID T-B+NK+	died	+	

* Exclusion of Rag_1_, Rag_2 _and Artemis defect

CMID = cell mediated immune defect, CGD = chronic granulomatous disease, SCID = severe combined immunodeficiency, DLN = distal lymph node.

Comparison of immunologic markers showed that significant differences were seen between two groups of children. The mean value of CD3 in patients was (P = 0.005) in comparison with control group and the mean value of CD4 in patients was (P < 0.001) in comparison with control group (Table 3).

**Table 3:** The mean of immunologic markers in patients and control groups

Immunologic markers	Patients (%) Mean ± SD	Control (%) Mean ± SD	P. value
CD3	30.82 ± 28.98	62.11 ± 8.77	0.005
CD4	13.36 ± 14.21	42.12 ± 11.34	0.001
CD8	19.00 ± 13.61	22.30 ± 6.06	0.452
CD19	23.91 ± 15.40	21.19 ± 8.26	0.586
CD56	35.18 ± 30.46	10.57 ± 4.90	0.029

Although antimycobacterial regimens used for the treatment of patients with 4 drugs, but 8 of the patients died despite aggressive management.

## Discussion

BCG is a live attenuated bacterial vaccine that protects children from miliary tuberculosis and tuberculosis meningitis [10]. It is considered a safe vaccine with a low incidence of complications, such as purulent lymphadenitis and Bone BCG infection [2,3,9,11,12]. A disseminated infection is even rarer [6-8] but in recent years has increased up 0-1/100000 vaccinated children [6-8,13].

Lotte et al identified 60 cases of dissemination for which the mortality rate was %50 and cellular immunodeficiency was identified as the chief risk factors for fatal outcome [14,15]. Additionally the estimated incidence of disseminated disease in the 5.5 million vaccinated infants in six European countries was 2 cases per 1 million vaccinated children, and the mortality rate was 80% [14]. Disseminated BCG infections have occurred following vaccination of children with immunodeficiency disorders but only rarely in apparently normal individuals [6,8-10,16-20].

Also in 28 cases of definite disseminated BCG disease in 13 countries; immune defects were identified in 24(86%) of cases. Of 20 patients who died of disseminated BCG disease, all had an immune defect whereas the mortality rate among patients without an identified immunodeficiency was zero [6]. In Canada, 21 BCG vaccinated child with adverse reactions have detected with 6 disseminated BCG infection which 5 of them were died [21].

In our study 2/3 of our patients with disseminated BCG infection, had immunodeficiency that all of them died.

Our results are compatible with our pilot study that was done in children's hospital of Tabriz. In this study we could identify 8 cases of immunodeficiency that all of them had severe combined immunodeficiency and died due to disseminated BCG infection with mortality rate100% (in press). Also in retrospective study of 17 cases with disseminated BCG in Tehran impaired immunity was detected in 10 cases that all of them died [22].

Complications of BCG vaccine usually develops 5 months to 5 years after vaccination [6,9,22]. Also review over 5000 reports showed that 71%(20) of 28 cases occurred in patients younger than 2 years of age [6]. In our study, except 2 cases that were within 3 years old, all of them were younger than one year old.

The most commonly reported symptoms in the definite cases of disseminated BCG disease were fever, diarrhea, cough, Lymphadenopathy and weight loss, FTT and Hepatosplenomegaly, were also common [6,15,19,22,23]. In our study fever was in 100% and oral Candida was in 81.8%. Also thymus atrophy in 81.8% of patients was found. Anemia was the most common sign in our patients which was found in 100% of patients.

On the other hands, more than half (7 of 11 cases) of our patients had related parents which is compatible with study of Casanova et al. that parental consanguinity was found in 30% of the families [8] and also in study by Afshar et al. that related parents was found in 82/35% of patients [22].

## Conclusion

AT present BCG inoculation is necessary in countries with high incidence of tuberculosis, but has a high mortality in patients with primary immunodeficiency, so it seems to make an effort in development of a more safer, inoffensive vaccine with minimum side effects are a necessity because infants with immunodeficiency are vaccinated at birth prior to diagnosis.

On the other hands, inoculation of BCG vaccine should be prohibited for a few months in the families with history of inherited immunodeficiency. Also, screening of newborns at birth for SCID especially in region with high incidence of SCID such as in the North West Iran [24] can be useful for identify high risk children.

Finally BCG lymphadenitis with concomitant signs and symptoms that we mentioned in our study, are requisite for evaluation of underlying disorders such as immunodeficiency.

## Competing interests

The authors declare that they have no competing interests.

## Authors' contributions

Ms sh: examination of patients, diagnosis and treatment of them, and corresponding author, KA: Head of TB and lung disease research center and supporting by a grant from the TB and lung disease research center, help in drafting the proposal, SM: laboratory analysis (flowcytometry) of samples, MR: participate in draft the manuscript, ZP: analysis of data, RB: examination of patients, AK: the manager of public health center, referral of cases, JC: involving in genetic studies, participate in the design of the study and revising it, JF: involving in genetic studies of Samples, JV: involving in genetic studies of samples. All authors read and approved the final manuscript.
